# Metformin treatment in diabetes and heart failure: when academic equipoise meets clinical reality

**DOI:** 10.1186/1745-6215-10-12

**Published:** 2009-02-09

**Authors:** Dean T Eurich, Ross T Tsuyuki, Sumit R Majumdar, Finlay A McAlister, Richard Lewanczuk, Marcelo C Shibata, Jeffrey A Johnson

**Affiliations:** 1 School of Public Health, University of Alberta, Edmonton, Alberta, T6G 2G3, Canada; 2 Faculty of Medicine and Dentistry, University of Alberta, Edmonton, Alberta, T6G 2C7, Canada

## Abstract

**Objective:**

Metformin has had a 'black box' contraindication in diabetic patients with heart failure (HF), but many believe it to be the treatment of choice in this setting. Therefore, we attempted to conduct a pilot study to evaluate the feasibility of undertaking a large randomized controlled trial with clinical endpoints.

**Study Design:**

The pilot study was a randomized double blinded placebo controlled trial. Patients with HF and type 2 diabetes were screened in hospitals and HF clinics in Edmonton, Alberta, Canada (population ~1 million). Major exclusion criteria included the current use of insulin or high dose metformin, decreased renal function, or a glycosylated hemoglobin <7%. Patients were to be randomized to 1500 mg of metformin daily or matching placebo and followed for 6 months for a variety of functional outcomes, as well as clinical events.

**Results:**

Fifty-eight patients were screened over a six month period and all were excluded. Because of futility with respect to enrollment, the pilot study was abandoned. The mean age of screened patients was 77 (SD 9) years and 57% were male. The main reasons for exclusion were: use of insulin therapy (n = 23; 40%), glycosylated hemoglobin <7% (n = 17; 29%) and current use of high dose metformin (n = 12; 21%). Overall, contraindicated metformin therapy was the most commonly prescribed oral antihyperglycemic agent (n = 27; 51%). On average, patients were receiving 1,706 mg (SD 488 mg) of metformin daily and 12 (44%) used only metformin.

**Conclusion:**

Despite uncertainty in the scientific literature, there does not appear to be clinical uncertainty with regards to the safety or effectiveness of metformin in HF making a definitive randomized trial virtually impossible.

**Trial registration:**

ClinicalTrials.gov Identifier: NCT00325910

## Background

Metformin has been approved for use in the treatment of type 2 diabetes mellitus for nearly 3 decades in Europe and Canada and for a decade in the United States (US). Numerous studies have shown metformin to be highly effective and safe in the treatment of type 2 diabetes [1-3]. Metformin is the only antidiabetic agent that has been shown to reduce mortality in patients newly diagnosed with type 2 diabetes and the only antidiabetic agent not shown to be associated with increased morbidity and mortality in patients with cardiac disease, including heart failure [2-5].

Despite this, product labeling for metformin in Canada, the US, and the EU state that it is contraindicated in most patients with heart failure, although recent changes have been made in the US [6-8]. Diabetes is a common comorbidity in patients with heart failure and portends a particularly poor prognosis[9,10]. As such, significant proportion of patients with diabetes may be potentially denied a beneficial treatment. It is thus not surprising that there has been a vigorous debate in the literature about whether or not metformin should be used in patients with heart failure and type 2 diabetes[4,7,11-24].

Indeed, a recent systematic review and meta-analysis suggests that in patients with comorbid heart failure and diabetes, metformin is the only agent which has not been associated with harm[25]. Importantly, the current evidence supporting metformin's use in heart failure is based on two population-based epidemiologic studies and both recommended confirmation of their findings in randomized trials[4,17]. Given the robust debate in the literature about the role of metformin in heart failure, this appeared to be a question that needed to be resolved with a randomized controlled trial[26].

We designed and implemented a pilot study, PHANTOM (Patients with Heart Failure ANd Type 2 Diabetes Treated with Placebo Or Metformin), to evaluate the feasibility of a large randomized controlled trial of metformin in patients with heart failure and type 2 diabetes (*ClinicalTrials.gov Identifier: NCT00325910)*. This paper describes our experiences in the implementation of the PHANTOM Pilot study and its implications for other clinical trials.

## Methods

### Study Design

The PHANTOM pilot study was a multi-centre, prospective randomized blinded placebo controlled trial designed to examine functionality, morbidity, and mortality outcomes in patients with heart failure and diabetes mellitus who are treated with metformin therapy over a 6 month period. Two hospitals, the University of Alberta Hospital and the Misericordia Hospital, and one outpatient specialized heart failure clinic in Edmonton, Alberta, Canada (population 1 million) participated in the study[27]. The University of Alberta Hospital is an 800-bed tertiary university-based teaching hospital and has been a recruitment site for many major cardiovascular trials including the Digitalis Investigation Group (DIG) Trial, the Heart Outcomes Prevention Evaluation (HOPE) Study, the Studies of Left Ventricular Dysfunction (SOLVD), the Global Utilization of Streptokinase and Tissue Plasminogen Activator for Occluded Coronary Arteries (GUSTO) trials. The Misericordia Hospital is a 500-bed community hospital also involved in several large randomized trials in acute coronary syndromes and heart failure. The study protocol was reviewed and approved by the Research Ethics Board of all participating sites.

### Eligibility

We screened consecutive patients older than 18 years, admitted to the hospital or emergency room or registered patients of the local heart failure clinics. Inclusion criteria were: physician diagnoses of symptomatic heart failure (NYHA class II, III, IV) and type 2 diabetes (i.e., a previous physician diagnosis or actively receiving oral antidiabetic agents or a new diagnosis of type 2 diabetes, defined as a fasting blood glucose ≥ 7.0 mmol/L or random blood glucose ≥ 11.1 mmol/L, and accompanied by acute metabolic decompensation or 2 hour plasma glucose in a 75 gram oral glucose tolerance test ≥ 11.1 mmol/L)[28]. We excluded patients if they were receiving greater than 1500 mg of metformin daily, were unwilling to change their antidiabetic regimens, were receiving insulin therapy, had a serum creatinine ≥ 180 μmol/L, had an A1c < 7.0%, were unable to communicate because of language barriers, had dementia or mental illness, were unwilling to complete self-monitoring of serum blood sugars during the trial period, were participating in another heart failure or diabetes clinical trial involving medications, or had significant comorbidities or a terminal illness precluding them from following the trial protocol over the 6 month follow-up period.

### Screening

Potential patients were identified through referrals of hospitalized or heart failure clinic patients. After identification, a two-stage screening process began. In stage 1, potentially eligible individuals were screened for non-invasive inclusion/exclusion criteria (i.e., all exclusion criteria except A1c and serum creatinine). In stage 2, patients who were not excluded after stage 1 were approached for consent to a blood sample to determine A1c and serum creatinine eligibility (if not previously completed as part of their clinic or hospital medical care). Eligible patients meeting all inclusion and exclusion criteria and consenting to the trial continued with the study protocol.

### Randomization and study procedures

Consenting patients were to be randomized to either metformin or matching placebo tablets in a 1:1 ratio. Randomization was planned through a secure website provided by the project office (Epidemiology Coordinating and Research (EPICORE) Centre, University of Alberta).

Patients were to be assessed at baseline and six months later, with monthly telephone contact to assess response to the study medication and ascertain clinical outcomes. Follow-up visits and blood testing (A1c and serum creatinine) were planned for 3 and 6 month visits. We planned a dosage titration protocol where patients would be instructed to slowly titrate their study medication to a maximum dose of 1500 mg per day over a 3 week period based on a published titration protocol[1].

### Study Endpoints

The primary outcome was a composite endpoint of all-cause mortality or all-cause hospitalization. As secondary endpoints, comparisons of mean change in A1c, six minute walk distance, and mean change in health-related quality of life (i.e., RAND-12, EQ-5D, and Kansas City Cardiomyopathy Questionnaire) scores from baseline to the 6 month follow-up visit were also planned. In addition, we established criteria for monitoring safety of metformin, to identify patients developing lactic acidosis requiring urgent medical attention, as defined as an emergency room visit or hospitalization.

### Sample Size Considerations

We anticipated the full study would require a sample size of 1000 patients with heart failure and diabetes to detect an absolute difference in event rates of at least 10% based on an incidence rate of 50%[29] in our primary outcome per year with a two-tailed alpha = 0.05 and beta = 0.10. The goal of the pilot study was to enroll 100 patients.

## Results

Recruitment for the study began May 1, 2006. As of October 15^th^, 2006, fifty-eight consecutive patients with diabetes and heart failure were screened through the outpatient ambulatory heart failure clinic (n = 8, 14%) and in-patient cardiology and general medical wards (n = 50, 86%) with no patients meeting eligibility criteria. Using a conservative estimate of 1 patient enrolled for every 10 patients screened, our data suggests that the likelihood that a significant number of potentially eligible patients were missed during screening would be less than 5%. Indeed, review of the Regional Diabetes Program electronic databases, which at the time contained approximately 500 individuals with type 2 diabetes and heart failure, suggests very few patients (<1%) would have been *potentially *eligible for the trial. This poor availability of suitable patients led the Steering Committee to recommend that the study be abandoned.

The characteristics of the individuals screened for the pilot study are shown in Table 1. Of note, 5 patients refused to have their screening data collected and are therefore not included in our results. Of the patients admitted to hospital (n = 45, 85%), heart failure (n = 22, 49%) was the most common admitting diagnosis. Acute coronary syndromes (n = 7, 16%) and diabetes (n = 4 (9%) were the next most common reasons for admission. The median length of stay in hospital was 12 days (interquartile range 9 to 36).

**Table 1:** Clinical characteristics of screened patients

Characteristic*	**Overall Group** (n = 53)*	**Non-Metformin User** **(n = 26)**	**Metformin User** **(n = 27)**	**P-value‡**
		**No. (%) or Mean ± SD**		
			
Age – yrs	76.5 ± 8.6	78.2 ± 8.5	74.9 ± 8.7	0.17
Sex – male	30 (57)	16 (62)	14 (52)	0.48
Weight (kg)	82 ± 25	80 ± 22	84 ± 28	0.74
Serum Creatinine (μmol/L)	135 ± 64	147 ± 75	123 ± 49	0.17
Creatinine Clearance (ml/min)	49 ± 23	45 ± 18	53 ± 26	0.19
A1c (%)^†^	7.3 ± 0.02	6.8 ± 1.2	7.7 ± 2.6	0.22
Heart Failure Medications				
Beta-Blockers	38 (72)	18 (69)	20 (74)	0.70
ACE Inhibitors or ARBs	51 (96)	26 (100)	25 (93)	0.49
Calcium Channel Blockers	18 (34)	8 (31)	10 (37)	0.63
Antiplatelet Agents	44 (83)	21 (81)	23 (85)	0.73
Digoxin	9 (17)	4 (15)	5 (19)	1.0
Spironolactone	12 (23)	5 (19)	7 (26)	0.56
Lipid Therapy	41 (77)	19 (73)	22 (82)	0.47
Nitrates	10 (19)	4 (15)	6 (22)	0.73
Diabetes Medications				
Insulin	23 (43)	15 (58)	8 (30)	0.039
Sulfonylureas	8 (15)	3 (12)	5 (19)	0.70
Thiazolidinediones	4 (8)	1 (4)	3 (11)	0.61
Meglitinides	2 (4)	1 (4)	1 (4)	1.00

* – five patients refused to have data included

† – 34 people had A1c assessed in previous 3 months; 15 people in non-metformin user and 19 people in metformin user groups

**‡ **– for comparison between non-metformin and metformin group

Overall, the patients screened represented a typical population of patients with heart failure with 57% males and an average age of 77 (SD 9) years. At the time of screening, 51 (96%) patients were receiving an angiotensin converting enzyme (ACE) inhibitor or angiotension receptor blockers (ARB) (Table 1). Approximately two-thirds of patients were also receiving beta-blockers, antiplatelet and lipid-lowering therapies with 27 (51%) patients receiving all three therapies.

**Figure 1:**
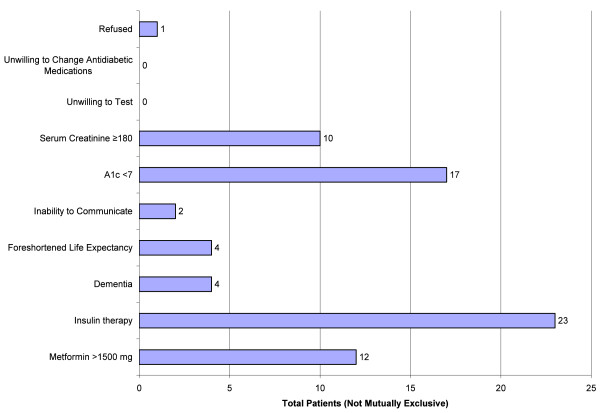
**Reason for study exclusion**.

The use of insulin (n = 23, 43%) was the most common reason for trial exclusion (Figure 1). Of the patients receiving insulin therapy, 11 (48%) had additional exclusion criteria. Overall, 11 (48%) patients received insulin in combination with oral antidiabetic agents. The second most common reason for exclusion from the trial was an A1c value less than 7%. Of the patients who had an A1c available (n = 35, 65%), 17 (49%) patients were excluded with an A1c less than 7% reported in their medical records (Figure 1). The mean A1c was 7.3% (SD 2) (Table 1). This may misrepresent the true A1c values, however, as not all patients had an A1c measured due to the two stage screening protocol.

Despite being 'absolutely' contraindicated in this population during our period of study, metformin was the most commonly prescribed oral antidiabetic agent with 27 (51%) patients receiving it at the time of screening (Table 1). In comparison, few patients were receiving therapy with sulfonylureas, thiazolidinediones, meglitinides or alpha glucosidase inhibitors (Table 1). Although the duration of therapy with metformin in these patients was not ascertained, 4 subjects with heart failure were newly initiated on metformin during their hospitalization. Of the patients receiving metformin, 12 (44%) were receiving greater than 1500 mg per day and were therefore excluded from our study (Figure 1). On average, patients were receiving 1,706 mg per day (SD 488) with 2000 mg per day as the most common daily dose regimen. Twelve (44%) patients used metformin as monotherapy, 9 (33%) in combination with other oral agents and 6 (22%) in combination with insulin alone. Metformin was also more commonly prescribed in combination with insulin than other oral agents. Of the 23 patients receiving insulin therapy, 8 (35%) were also prescribed metformin. We saw no significant differences between patients receiving metformin and non-metformin regimens in terms of clinical or demographic characteristics (Table 1).

## Discussion

A guiding principle for the conduct of a randomized controlled trial is that of equipoise, or perhaps more appropriately 'uncertainty' as to whether the therapy under study works[26]. Although metformin has been demonstrated to improve outcomes, its use in people with heart failure and type 2 diabetes is still a contentious issue. Clinicians and researchers have argued both sides of the issue providing strong evidence for the criterion of uncertainty[4,7,11-24]. Results of our pilot study would suggest, however, that there may be discordance between the scientific community and practicing clinicians.

Over 50% of patients with diabetes and heart failure in our pilot study were receiving metformin, either alone or in combination with other antidiabetic agents, suggesting there is no uncertainty in the eyes of clinicians. Although this may be surprising, one must consider that metformin therapy is one of the most commonly prescribed antidiabetic agents and has a long history of use in Canada. Indeed, a recent population based study using Canadian data suggested that two-thirds of all patients with type 2 diabetes treated with antidiabetic medications receive metformin therapy, either alone or in combination with other agents[30]. Furthermore, observational data in Canada, Europe, and the US have shown that 20 to 25% of patients receiving metformin therapy also have comorbid heart failure[4,11,17,20,23,31,32] and US data has indicated that the use of metformin has increased by over 50% in patients with heart failure in recent years[12]. In our pilot study, half of all subjects were actively receiving metformin therapy with the majority of people receiving relatively high doses of metformin therapy (i.e., >1500 mg/day). Given the accumulated research, including our pilot data, it would seem there is little uncertainty at the clinician level regarding the use of metformin in patients with heart failure.

Should there be uncertainty with regards to the use of metformin in patients with heart failure? Perhaps not, considering the evidence base for the efficacy of metformin in type 2 diabetes. Indeed, numerous observational and randomized controlled studies have clearly shown metformin to be highly efficacious for patients with type 2 diabetes[1-4,17]. Moreover, epidemiological evidence has suggested that metformin may be associated with improved outcomes in patients with heart failure[4,17,25]. The potential scientific uncertainty with using metformin in patients with heart failure seems to be focused on the risks rather than the possible benefits. One should note, however that although there are two large meta-analyses and a case series indicating that the risk of lactic acidosis with metformin is an extremely rare event occurring at the same rate as other antidiabetic agents, uncertainty around its safety suggested that a randomized controlled trial was warranted. This uncertainty, however, seems to be waning.

Since the implementation of the pilot study, several fundamental changes have occurred surrounding the use of metformin in both Canada and the United States. In 2007, the United States Food and Drug Administration removed the heart failure contraindication from the packaging label for metformin therapy (Glucophage^® ^and Glucophage XR^®^); however, a *'black-box' warning *still exists for the use of metformin in this population suggesting caution if it is used at all. Similar labeling changes have not yet occurred in Canada, although the 2008 Canadian Diabetes Association clinical practice guidelines have indicated that metformin should be considered as first-line therapy in patients with heart failure (Grade C, Level 3 [observational evidence])[33].

Our experience should be viewed in light of several other considerations. The inability to enroll patients into the pilot study may have been influenced by numerous factors including the patient-clinician relationship and the perceived importance of the trial[34]. It is possible, for example, that admitting the potential uncertainty of using metformin in heart failure by the clinician may be perceived as damaging to the patient-clinician relationship; thereby becoming a barrier to patient enrollment[35,36]. Although all clinicians directly involved in our study were supportive, those outside of the study may not have believed the research question was of interest or importance[37]. It is also possible that despite the contraindication at the time, individual clinicians prescribing for these patients felt that the benefits outweighed any potential risks, and may have felt uneasy with the potential withdrawal of metformin therapy. If this is indeed the case, even if a successful randomized control trial was conducted, one must ask "will it substantially alter prescribing patterns of the front line clinician?" Numerous examples exist in the literature where randomized controlled trials have changed the collective thinking of the scientific community but have failed to make significant impacts on the prescribing patterns of individual clinicians[38].

In addition, as with any clinical trial, the inclusion and exclusion criteria clearly affected potential enrollment. We feel, however, few modifications could be made to our already liberal inclusion criteria, requiring only a physician diagnosis of heart failure and type 2 diabetes. Previous metformin use was not an exclusion criteria but patients receiving high dose metformin therapy (>1500 mg/day) were excluded as it was considered unethical to potentially randomize patients to placebo for a medication previously deemed necessary by a physician. Similarly, patients with adequate control of their diabetes (i.e., A1c <7%) were also excluded as it was deemed potentially unsafe to initiate another antidiabetic therapy. With respect to renal function, the current product monograph recommends that metformin be avoided in patients with a creatinine clearance <60 mL/min[6]. In our study, the criteria was considerably more liberal, allowing patients with renal function as low as 20–30 mL/min. Of note, the use of metformin in the setting of chronic kidney disease with glomerular filtration rates less than 60 ml/min is also a major contraindication where labeling remains an issue.

Lastly, patients using insulin therapy were excluded. Previous research has suggested that insulin therapy may be associated with an increased risk of heart failure[10,39] and also an increased risk of mortality in people with diabetes and heart failure[5,40,41]. We therefore felt it was important to exclude this treatment to focus on the effects of metformin. Even if we had not excluded insulin users, 48% of these patients would not have been eligible due to other exclusions. As a result, changes in the inclusion and exclusion criteria would likely have not significantly altered our study outcome.

## Conclusion

Our pilot data, coupled with the recent legal and clinical guideline endorsement for the use of metformin in heart failure patients, suggest that any future large randomized controlled trials with metformin in patients with heart failure and diabetes will be an arduous undertaking. Given the already frequent and increasing clinical experience of using metformin in patients with heart failure, a trial design which limits the use of metformin therapy may have difficulties in gaining clinician commitment as clinical uncertainty does not appear to exist[26]. If safety is still the true concern, it is well known that randomized controlled trials are, by design, ill suited to address the issue of safety, especially when rare events are concerned[42,43]. As a result, a large, well designed, phase 4, prospective evaluation of metformin use in patients with heart failure may provide the best evidence[42,43].

## Competing interests

The authors declare that they have no competing interests.

## Authors' contributions

All authors were involved in the conception and design and acquisition of data. DTE analyzed the data and drafted the first manuscript. All authors were involved in the interpretation of the data and revised it critically for important intellectual content. All authors read and approved the final manuscript.

The funding sources had no role in the design and conduct of the study; the collection, analysis, interpretation of the data, or in the decision to submit the manuscript for publication.
